# Trbp inhibits cardiac fibrosis through TGF-β pathway-mediated cross-talk between cardiomyocytes and fibroblasts

**DOI:** 10.1042/CS20242397

**Published:** 2025-03-11

**Authors:** Bo Pan, Di Hu, Yao Wei Lu, Jing Luo, Xiao Hui Xu, Haipeng Guo, Rui Deng, Zhuomin Liang, Yi Wang, Qing Ma, John D. Mably, Jie Tian, Da-Zhi Wang

**Affiliations:** 1 Department of Pediatric Cardiology, National Clinical Key Cardiovascular Specialty, Children’s Hospital of Chongqing Medical University, National Clinical Research Center for Child Health and Disorders, Ministry of Education Key Laboratory of Child Development and Disorders, Chongqing, China; 2 China International Science and Technology Cooperation Base of Child Development and Critical Disorders, Chongqing, China; 3 Key Laboratory of Children’s Important Organ Development and Diseases of Chongqing Municipal Health Commission, Chongqing, China; 4 Department of Cardiology, Boston Children’s Hospital, Harvard Medical School, Boston MA 02115, U.S.A; 5 Department of Otorhinolaryngology, Children’s Hospital of Chongqing Medical University, Chongqing, China; 6 The Center for Regenerative Medicine and USF Health Heart Institute, Morsani College of Medicine, University of South Florida, Tampa, FL 33602, U.S.A; 7 Department of Internal Medicine, USF Health, University of South Florida, Tampa, FL 33602, U.S.A; 8 Department of Molecular Pharmacology & Physiology, USF Health, University of South Florida, Tampa, FL 33602, U.S.A

**Keywords:** cardiac fibrosis, cross-talk, heart failure, TGF-β, Trbp

## Abstract

Cardiac remodeling in response to disease or tissue damage severely impairs heart function. Therefore, the description of the molecular mechanisms responsible is essential for the development of effective therapies. Trbp (Tarbp2) is a multifunctional RNA-binding protein that is essential during heart development, but its role in the adult heart and cardiac remodeling remains unknown. We generated inducible conditional knockout mice to delete Trbp from cardiomyocytes in young adults (Trbp-cKOs). While Trbp-cKO mice did not display a detectable phenotype, under stress conditions induced by transverse aortic constriction pressure overload, they rapidly developed severe heart failure; this was associated with maladaptive cardiac remodeling and increased interstitial fibrosis. RNA-sequencing revealed the induction of a fibrotic gene expression network and the TGF-β signaling pathway in Trbp-cKO hearts. In cultured neonatal rat ventricle cardiomyocytes (NRCMs), inhibition of Trbp resulted in an induction of the expression of both *Tgfβ2* and *Ltbp2*; in contrast, Trbp overexpression repressed *Tgfβ2* expression. Knockdown of Trbp in NRCMs that were co-cultured with neonatal rat cardiac fibroblasts (NRCFs) resulted in an increase in fibrotic gene expression. However, knockdown of Trbp in NRCMs combined with knockdown of *Tgfβ2* in NRCFs using the same co-culture system failed to induce the same change in fibrotic gene expression. These data provide evidence for a critical role for Trbp in regulating cardiac fibrosis during cardiac remodeling mediated by cross-talk between cardiomyocytes and fibroblasts. The link to TGF-β signaling also highlights its importance and reveals a novel approach to intervention by targeting of Trbp.

## Introduction

The morphogenesis of the heart is associated with significant changes in gene expression patterns. During the transition from fetal to adult, cardiomyocytes demonstrate a switch in sarcomeric gene isoform expression, as well as changes in metabolic signaling pathways and ion transport systems [1,2]. Trbp, also known as Tarbp2, was initially identified as an RNA-binding protein (RBP) involved in HIV pathogenesis [3] but has also been linked to cell growth processes and cancer [4]. Additional studies have shown it to be a crucial regulator of many cellular functions; in addition to its role in HIV replication, it is involved in the regulation of the IFN and stress response pathways, sequestration of the protein PACT, and is an essential component of the RNA interference (RNAi) machinery [5–7].

We previously generated and described a Trbp cardiac conditional knockout mouse model using cTNT-cre to investigate its role during heart development [8]. Our previous studies demonstrated that in embryonic and postnatal hearts, loss of Trbp severely impaired cardiac contraction through alteration of the balance between slow- (e.g., decreased Myl3, Myh7b) and fast-twitch (e.g., increased Myl9, Tnni2) muscle fiber gene isoforms [8]. However, whether Trbp plays a similar role in the regulation of the sarcomeric protein isoforms in the mature heart remains unknown; furthermore, the mature heart may not display a substantial functional change since it may be less susceptible to the effects of such isoform expression changes than the developing heart.

The TGF-β superfamily is a well-characterized signaling pathway that has been implicated in the regulation of fibrosis [9,10]. Most cells express receptor complexes that transduce TGF-β signaling; signal specificity is achieved by spatial control, through the localized secretion of ligands. The ligand is secreted alongside the latency-associated peptide (LAP) and latent TGF-β-binding protein 1–4 (LTBP1–4) complexes; these serve to sequester the ligand in the extracellular matrix until it is able to escape the LAP and LTBP complex [11,12]. Only then the ligand is able to bind TGF-β receptors on target cells. The expression of these ligands is sufficient to induce proliferation in some specific cell types such as fibroblasts and smooth muscle cells.

The objective of the present study was to determine the role of Trbp in mature hearts. We first deleted Trbp in cardiomyocytes in young adult mice and did not observe a detectable phenotype; however, we did observe an increase in the TGF-β ligand genes *Tgfβ2* and *Ltbp2*. Transverse aortic constriction (TAC) surgery was subsequently performed on these animals to induce cardiac hypertrophy and determine the role of Trbp under stress conditions. We found that stressing the hearts from Trbp cardiomyocyte-specific inducible knockout (Trbp-cKO) animals induced changes in the expression of fibrotic network genes (e.g., *Col1a1*, *Col3a1*, *Col5a2*, and *Postn*) alongside continued increases in *Tgfβ2* and *Ltbp2*. Using a co-culture system, we showed that Trbp plays a critical role in regulating cardiac fibrosis by mediating the cross-talk between cardiomyocytes and fibroblasts through the TGF-β signaling pathway. Our data indicate that in adults, Trbp plays an important role in the inhibition of cardiac fibrosis through the regulation of the TGF-β pathway-mediated cross-talk between cardiomyocytes and fibroblast cells.

## Materials and methods

### Laboratory animal use

All animal experiments were performed according to protocols approved by the Institutional Animal Care and Use Committees of Boston Children’s Hospital and the University of South Florida. All procedures conform to the guidelines in the NIH Guide for the Care and Use of Laboratory Animals. The rodents were killed by carbon dioxide inhalation in accordance with AVMA guidelines. Flow rates of 4 and 10 l/min of CO_2_ were used for mice and rats, respectively, due to the difference in cage sizes. Following the procedure, animals were observed for a minimum of 10 min to ensure cessation of cardiovascular and respiratory movements. For killing rodent neonates aged P8–P10, CO_2_ inhalation followed by cervical dislocation as a secondary method was used. Similarly, for rodents older than P10 and under 200 g, CO_2_ inhalation was used followed by cervical dislocation or decapitation as a secondary method. Since rodent neonates (up until approximately 10 days) are resistant to killing by CO_2_, the killing of day 1 rat pups for the preparation of neonatal rat ventricle myocytes (NRVMs) and neonatal rat cardiac fibroblasts (NRCFs) was performed by cervical dislocation. In all cases, death was verified prior to carcass disposal.

### Mouse line generation

Trbp-flox/flox (Trbp-fl/fl) mice were generated as previously described [8]. The alpha-MHC-MerCreMer (αMHC-MCM) transgenic mice were purchased from the Jackson Lab and bred with Trbp-fl/fl mice to obtain Trbp-fl/fl-MCM mice. Tamoxifen (TAM) (30 mg/kg) was injected once daily for five days by intraperitoneal injection into four-week-old male Trbp-fl/fl-MCM mice to obtain Trbp-cKO mice. Corn oil was injected as vehicle control into four-week-old male Trbp-fl/fl-MCM mice. Trbp-fl/fl male mice were also injected with TAM (30 mg/kg for five consecutive days) to control for the potential side effects of TAM.

### TAC

Mice were anesthetized with isoflurane delivered using a scavenger-equipped calibrated vaporizer. Induction was performed in an induction chamber with an initial vaporizer setting of 3–5%. Animals were removed from the chamber after induction and placed on a rodent-specific nose cone apparatus with maintenance anesthesia provided using an isoflurane vaporizer setting of 1–2%. The chest was shaved and cleaned with alcohol. A suture was placed around the front upper incisors and pulled taut, so that the neck was slightly extended. The tongue was retracted and held with forceps, and a 20-gauge catheter was inserted into the trachea. The catheter was then attached to the mouse ventilator via a Y-shaped connector. Ventilation was performed with a tidal volume of 220–240 μl for a 25–30-g mouse and a respiratory rate of 130–140 breaths per minute. 100% oxygen was provided to the inflow of the ventilator. The chest was opened through a left second intercostal thoracotomy. The 26-gauge needle without its sharp tip was placed on the ascending aorta. The needle and the ascending aorta were tightly ligated together using a 7–0 nylon suture (Ethicon) at the position between the brachiocephalic artery and the common carotid artery, and the 26-gauge needle was removed immediately after ligation. In the sham operation, no ligation was performed. Isoflurane was stopped, and the lungs were slightly overinflated to assist in the removal of air in the pleural cavity. Dissected intercostal space and chest skin were closed using a 6–0 silk suture (Ethicon). All manipulations were performed by an operator without the knowledge of the genotype.

### Echocardiography

Two-dimensional (2-D) and M-mode imaging was performed using a VisualSonics Vevo 2100 Imaging System with a 40-MHz MicroScan transducer (model MS-550D) as previously described [4,8]. Mice were anesthetized with isoflurane (2.5% isoflurane for induction and 0.1% for maintenance). Heart rate and left ventricular (LV) dimensions were measured from 2-D short-axis under M-mode tracings at the level of the papillary muscle. Functional parameters such as percentage of ejection fraction (EF%), fractional shortening (FS%), and LV mass were calculated using the above primary measurements and accompanying software.

### Histology

Mouse hearts were dissected out, rinsed with PBS, and arrested in diastole with KCl and BDM buffer, then fixed in 4% paraformaldehyde (pH 7.4) overnight. After dehydration through a series of ethanol baths, samples were embedded in paraffin wax according to standard laboratory procedures. Sections of 5 μm were stained with hematoxylin and eosin (H&E), or further fixed with prewarmed Bouins’ solution, 55°C for 1 h, and stained with Fast Green as previously described [4,13]. The stained sections were used for routine histological examination with light microscope and quantified with ImageJ software.

### RNA-sequencing and data analysis

RNA from mouse hearts approximately eight weeks after either sham or TAC surgery was prepared for gene expression profiling (four biological replicates for each group). Total cardiac RNAs were isolated from fresh ventricular tissue using TRIzol (ThermoFisher Scientific) and were twice oligo(dT)-selected using the Dynabead mRNA purification system (Invitrogen). The RNA samples were quantified and assessed for quality prior to being sent to GENEWIZ for RNA-sequencing (RNA-seq). The RNA-seq was processed by GENEWIZ using Illumina platform (paired-ended, 150 bp read-length). FASTQ files were extracted, and the TruSeq sequencing adapters and low-quality reads were removed from FASTQ files with Cutadapt (v.2.3). The cleaned FASTQ files were quality checked using FastQC (Babraham Bioinformatics) and then aligned to the genome (GRCm38 obtained from GENCODE) using HISAT2 (v.2.1.0). Subsequently, transcript assembly was performed using StringTie (v.1.3.4) with the annotated transcriptome as a reference. The assembled transcriptomes were quantified using prepDE.py script provided by the StringTie developer to generate gene matrix files. EdgeR (v.3.26.1) was used to compute counts per million as a normalized measurement for gene expression. Differentially expressed genes were tested using the Fisher’s exact test, and multiplicity correction is performed with the Benjamini–Hochberg method on the *P* values, to control the false discovery rate (FDR). Filtering conditions: genes differentially expressed in each group were filtered by |logFC| ≥= 0.8, FDR ≤= 0.05.

### Total RNA extraction and quantitative RT-PCR

Total RNAs were isolated using TRIzol reagent (ThermoFisher Scientific) from cells and tissue samples. For quantitative RT-PCR detecting the expression of genes, 1 μg of RNA samples was reverse-transcribed to cDNA using random primers and MMLV reverse transcriptase (Invitrogen) in a 20-μl reaction system. The obtained cDNA samples were 10× diluted in nuclease-free water. For each reaction, 1 μl of diluted cDNA was used with SYBR Green and normalized to Gapdh. Sequences of PCR primers used in this study are listed in Supplementary Table 3.

### Western blot

Western blot was carried out as previously described. Briefly, protein lysate samples were prepared from heart tissues in RIPA buffer with proteinase inhibitors. Lysate samples (15–20 μg of total protein for each) were separated by 12% SDS-PAGE and electrophoretically transferred to PVDF membranes. Trbp protein was detected with Rabbit anti-Trbp antibody (AbCam, #42018). Collagen type I protein was probed with Rabbit anti-Collagen I (Proteintech, #14695–1-AP), and collagen type III protein was detected with Rabbit anti-Collagen III (Proteintech, #22734–1-AP). β-tubulin (mouse anti- β-Tubulin, Sigma, #T8328) and GAPDH (mouse anti-GAPDH, Origene, #TA802519) were used as loading controls for different protein analysis. Protein bands were visualized with ImageQuant LAS4000 image system.

### Isolation, culture, and treatment of NRVMs and NRCFs

NRVMs and NRCFs were isolated from day 1 rat pups using enzymatic digestion. All cells were cultured in DMEM supplemented with 10% FBS (Hyclone, New Zealand), maintained at 37°C in a humidified atmosphere containing 5% CO2. The Transwell Permeable Supports (Labselect, Beijing, China) with a 0.4-μm polycarbonate membrane were used in the co-culture model system to separate NRVMs and NRCFs into different compartments. 1.5 × 10^6^ NRVM cells in 1.5 ml of DMEM were seeded into the top chamber of a transwell insert. Four hours prior to co-culture, 2 × 10^5^ NRCF cells in 2.6 ml of DMEM were grown in six-well plate. Then, the transwell insert with transfected NRCMs was placed directly on top of the six-well plate containing the NRCFs. After incubation for the indicated time, NRCF cells in the lower layers were harvested for further analysis.

### RNAi assays

Synthetic siRNA oligonucleotides specific for Trbp: 5′-CGCAAAGAGUUCACCAUGACUTT-3′ and *Tgfβ2*: 5′-GGCUGAACAACGGAUUGAATT-3′ were synthesized by Shenggong (Shanghai, China) and Gene-Pharma (Shanghai, China), respectively. Stealth RNAi-negative control duplexes were used as negative controls (NCs). NRVMs were transiently transfected with Trbp and/or *Tgfβ2* siRNA or NC using RNAimax transfection reagent (Invitrogen, Carlsbad, CA, U.S.A.) according to the manufacturer’s instructions.

### Overexpression of Trbp

GV314Ad-Tarbp2 and GV314AD-GFP adenoviral vectors were purchased from Gene-Pharma Technology Co. Ltd. (Shanghai, China). NRVMs were plated onto slides in 24-well plates and allowed to reach 50–70% confluence at the time of transfection. The GV314Ad-GFP adenovirus was used as a control (NC). Adenoviral infection was performed according to the manufacturer’s instructions. NRVMs were incubated in growth medium with the adenoviruses at a multiplicity of infection of 40 for 2 h at 37°C and were then grown in new medium for another 36 h at 37°C.

### Statistics

Morphological and histological analyses were repeated at least three times. Four biological replicates were used for RNA-seq experiments. All other experiments have been repeated at least three times. Values are expressed as the mean ± SD. Statistical differences between groups were examined by unpaired Student’s *t*-test. The data were analyzed using GraphPad Prism5 software.

### Resource availability

The RNA-seq data are publicly available from the NIH NLM/NCBI GEO DataSets website (https://www.ncbi.nlm.nih.gov/gds) with the GEO accession number: GSE287292 [14]. Materials used in this study, including mouse lines, are available from the lead contacts upon reasonable request. Further information and requests for resources and reagents should be directed to Dr. Da-Zhi Wang (dazhiw@usf.edu) or Dr. Jie Tian (jietian@hospital.cqmu.edu.cn).

## Results

### Cardiomyocyte-specific Trbp deletion results in decreased cardiac function in young adult mice

**Figure 1:**
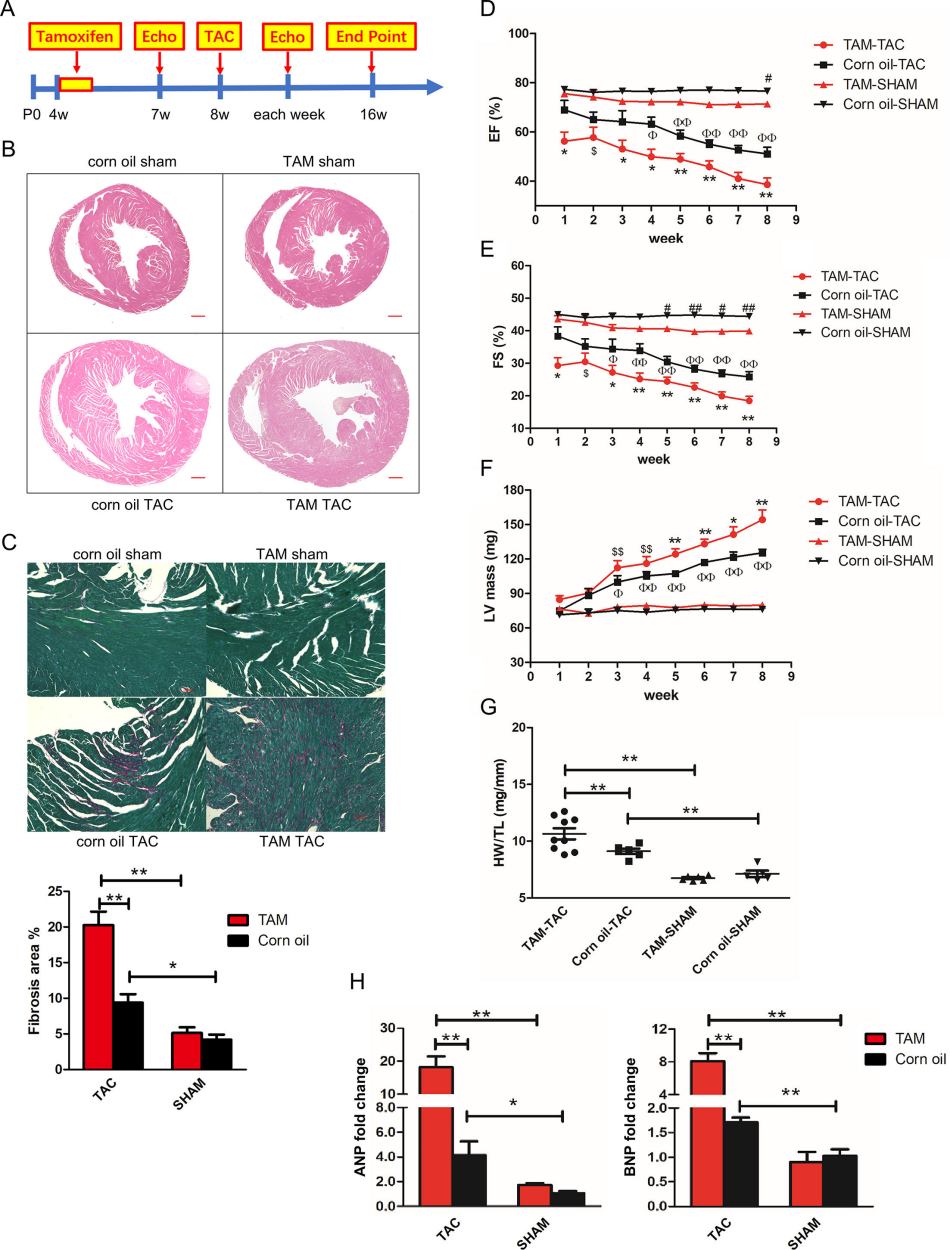
Influence of cardiomyocyte-specific deletion of Trbp on cardiac morphology and function in young adult mice. **(A)** Schematic representation of the timeline for analysis of Trbp deletion in young adult mice. TAC surgery was performed four weeks following TAM treatment. Echocardiography was performed prior to surgery, as well as at several stages after. (**B)** Representative images of cardiac sections stained with H&E from 16-week-old mouse hearts (8 weeks after TAC surgery). Scale bar = 500 µm. (**C**) Representative images of cardiac sections stained with Fast Green to highlight fibrotic regions from 16-week-old mouse hearts (8 weeks after TAC surgery). Scale bar = 100 µm. Quantification of fibrosis was measured as a percentage of total myocardial area in Trbp-cKO compared with control hearts at TAC surgical end point (TAM TAC, *n* = 4; corn-oil TAC, *n* = 4; TAM SHAM, *n* = 5; corn-oil SHAM, *n* = 4). *P < 0.05; **P < 0.01. (**D–F)** Assessment of ventricular function by echocardiography in Trbp-cKO mice compared with controls at indicated time points after TAC surgery (TAM TAC, 1–4w *n* = 10, 5–7w *n* = 9, 8 w *n* = 5; corn-oil TAC, 1–2w *n* = 7, 3–7w *n* = 6, 8 w *n* = 4; TAM SHAM, *n* = 6; corn-oil SHAM, *n* = 5). *TAM TAC vs. corn-oil TAC or TAM SHAM, *P < 0.05; **P < 0.01; ^$^TAM-TAC vs. TAM-SHAM, ^$^*P* < 0.05, ^$$^*P* < 0.01; ^#^Corn-oil SHAM vs. TAM-SHAM, ^#^*P* < 0.05, ^##^*P* < 0.01; ^Φ^corn-oil TAC vs. corn oil SHAM, ^Φ^P < 0.05, ^ΦΦ^*P* < 0.01. Parameters examined were** (D)** left ventricle ejection fraction (LVEF),** (E)** left ventricle fraction shortening (LVFS), and (**F)** left ventricle mass (LV MASS). (**G)** Heart weight-to-tibia length ratios (HW/TL) in Trbp-cKO and control mice eight weeks after TAC surgery; (**H)** qRT-PCR of ANP and BNP in Trbp-cKO hearts eight weeks after SHAM or TAC (ANP, TAM TAC, *n* = 7; corn-oil TAC, *n* = 6; TAM SHAM, *n* = 5; corn-oil SHAM, *n* = 5; BNP, TAM TAC, *n* = 4; corn-oil TAC, *n* = 3; TAM SHAM, *n* = 5; corn-oil SHAM, *n* = 4). *P < 0.05, **P < 0.01. Values are expressed as mean ± SD. **Figure 1C and 1H** : statistical differences between groups were examined by unpaired Student’s t-test. **Figure 1D, 1E and 1H** : statistical differences between groups were examined by one way ANOVA Newman−Keuls multiple comparison test. cKO, conditional knockout; TAC, transverse aortic constriction; TAM, tamoxifen.

To examine the functional role of Trbp in adult hearts, we generated a conditional Trbp-mutant mouse by breeding Trbp-flox (Trbp-fl/fl) mice with alpha-MHC-MerCreMer (αMHC-MerCreMer) mice. We started the induction by injecting TAM into four-week-old male Trbp-fl/fl, αMHC-MerCreMer mice (hereafter called Trbp-cKO mice); corn oil injected mice were used as controls (Figure 1A). We confirmed an 82.3% reduction in Trbp mRNA level in whole hearts one week after TAM injection (Supplementary Figure 1A) when compared with controls. The level of reduction is consistent with the reported recombination efficiency for this αMHC-MerCreMer strain [15,16]. Trbp protein showed a 63.5% reduction in the hearts of Trbp-cKO mice when compared with controls, consistent with the decreased transcript levels (Supplementary Figure 1B). No reduction in Trbp expression was observed in tissue from liver or skeletal muscle of Trbp-cKO mice, demonstrating the specificity of cardiac-specific Trbp deletion (Supplementary Figure 1C). General morphology and heart function of Trbp-fl/fl and Trbp-fl/fl-MCM mice were evaluated three weeks after TAM or corn oil injection. Since it has been reported that a high dose of TAM can cause transient cardiomyopathyy [17,18], we followed up heart function in mice receiving TAM injections by echocardiography. No significant changes were found in either Trbp-fl/fl and or Trbp-fl/fl-MCM male mice three weeks after TAM injection (Supplementary Table 1), indicating that TAM usage (30 mg/kg) was not inducing cardiac hypertrophy.

Baseline cardiac function was evaluated by echocardiography in 5- to 12-week-old Trbp-cKO and control mice (1–8 week after TAM injection). Staining with H&E (Figure 1B) and fast green (Figure 1C) demonstrated no obvious morphological changes and no significant cardiac fibrosis in the Trbp-cKO mice, respectively. A minor decrease in cardiac function was seen based on EF% (Figure 1D), FS% (Figure 1E), and LV mass (Figure 1F). However, heart weight-to-tibia length ratio (Figure 1G) was not altered in Trbp cKO mice confirming the absence of adverse cardiac remodeling at baseline after Trbp deletion. The heart function of Trbp-cKO and control mice was evaluated six months after TAM and corn oil injection, and a slight decrease in LVEF and LVFS was found in Trbp-cKO mice, but levels of those markers were still within normal ranges (Supplementary Table 2).

### Loss of Trbp accelerates cardiac maladaptation and remodeling in response to pressure overload

Next, we examined whether the loss of Trbp in adult hearts affects its response to stress. We subjected Trbp-cKO mice to TAC to induce pressure overload. Mice were followed up to eight weeks postsurgery or until they developed severe heart failure with clinical symptoms that required euthanasia (study end point). We measured cardiac function in anesthetized mice by echocardiography according to the schedule described in Figure 1A. We observed an accelerated decline in cardiac function (Figure 1D and E) and a more severe cardiac remodeling (Figure 1B, C and F) in Trbp-cKO mice after TAC compared with littermate controls. Meanwhile, ventricle weight-to-tibia length ratio was also significantly increased in Trbp-cKO mice after TAC (Figure 1G). Markers of heart failure, such as ANP and BNP, were also further increased in Trbp-cKO hearts after TAC (Figure 1H). Together, these results indicate that Trbp is required in adult heart to maintain its function in response to stress condition.

### Alteration of the expression of genes encoding fast- and slow-twitch myofibers in adult Trbp-cKO hearts

**Figure 2:**
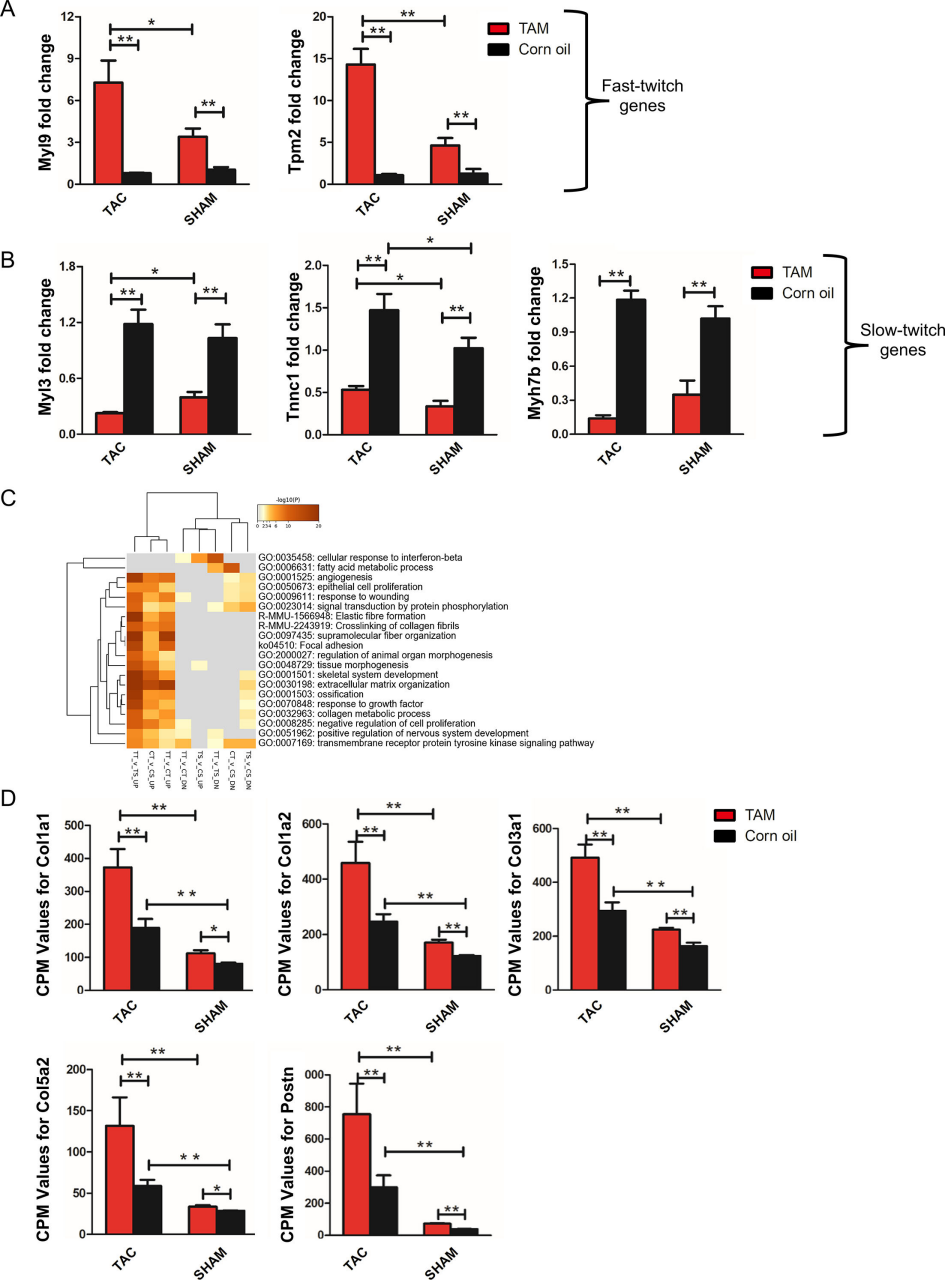
Trbp deficiency disrupts fast- and slow-twitch myofiber gene regulation and activates a fibrotic gene profile in the mature heart. qRT-PCR analysis of (**A)** fast-twitch and (**B)** slow-twitch myofiber gene expression in TrbpcKO hearts with or without TAC (For Myl9, Tpm2, Myl3 and Tnnc1: TAM TAC, *n* = 4; corn-oil TAC, *n* = 4; TAM SHAM, *n* = 5; corn-oil SHAM, *n* = 4. For Myh7b: TAM TAC, *n* = 6; corn-oil TAC, *n* = 6; TAM SHAM, *n* = 5; corn-oil SHAM, *n* = 5). (**C)** Enriched Ontology Clusters Among Groups classification of genes identified from RNA-seq analysis. Statistically enriched GO/KEGG terms were identified and cumulative hypergeometric *P* values, and enrichment factors were calculated and used for filtering. The remaining significant terms were then hierarchically clustered into a tree based on Kappa-statistical similarities among their gene memberships, and 0.3 kappa score was applied as the threshold to cast the tree into term clusters. (**D)** Counts per million values for fibrotic genes identified from RNA-seq data. *n* = 4, *P < 0.05; **P < 0.01. Values are expressed as mean ± SD. Statistical differences between groups were examined by unpaired Student’s *t*-test. cKO, conditional knockout; TAC, transverse aortic constriction; TAM, tamoxifen.

In our previous study [8], we found that deletion of Trbp altered fast- and slow-twitch myofiber gene expression during heart development, leading to an increase in levels of fast-twitch and decrease in levels of slow-twitch myofiber gene transcripts. The expression of these genes is precisely regulated during embryonic heart development and postnatal growth during which time the protein isoforms encoded by these genes are incorporated into cardiac sarcomeres; we extended these studies to inquire whether the deletion of Trbp in adult hearts could still affect the expression of these genes and, in turn, sarcomere structure. Similar to what was previously observed during development, we found that the loss of Trbp in cardiomyocytes of adult hearts results in a switch in the myofiber gene program (Figure 2A and B). In hearts without pressure overload, genes encoding fast-twitch muscle contractile proteins, including Tpm2 and Myl9, were significantly up-regulated in Trbp-cKO hearts (Figure 2A), while genes encoding Myl3, Tnnc1, and Myh7b (slow-twitch muscle contractile proteins) were dramatically down-regulated (Figure 2B). These results indicated that Trbp could still regulate sarcomeric isoforms switching in mature hearts, consistent with observations associated with Trbp-cKO during embryonic and postnatal stages.

### Increased expression of genes related to fibrotic and TGF-β signaling pathways in Trbp-cKO hearts

To understand the molecular mechanisms by which the loss of Trbp affects cardiac remodeling and function in young adult mice, we performed next-generation RNA-seq using tissue from hearts of Trbp-cKO mice and their littermates at eight-week post-TAC surgery (or sham). Multidimensional scaling plot shows that the transcriptome of the four groups is well segregated (Supplementary Figure 2A). Genes differentially expressed in each group were filtered by |logFC| ≥= 0.8, FDR ≤= 0.05; our analysis revealed that more genes were enriched in up-regulated pathways than down-regulated pathways (1652 up vs. 1092 down, Supplementary Figure 2B and C).

We then identified all statistically enriched GO/KEGG terms; cumulative hypergeometric *P* values and enrichment factors were calculated and used for filtering. The remaining significant terms were then hierarchically clustered into a tree based on Kappa-statistical similarities among their gene memberships. Then, 0.3 kappa score was applied as the threshold to cast the tree into term clusters (Figure 2C). Interestingly, 6 of 20 significantly up-regulated pathways were associated with the extracellular matrix (GO:0050673, GO:0009611, R-MMU-1566948, R-MMU-22243919, GO:0031098, and GO:0032963; Figure 2B). In the absence of pressure overload, fibrotic genes including *Col1a1*, *Col1a2*, *Col3a1*, *Col5a2*, and *Postn* were significantly up-regulated in young adult hearts with cardiomyocyte-specific loss of Trbp (Figure 2D). After TAC, the expression level of these fibrotic genes increased significantly more in hearts from Trbp-cKO mice than in the control TAC group (Figure 2D).

**Figure 3:**
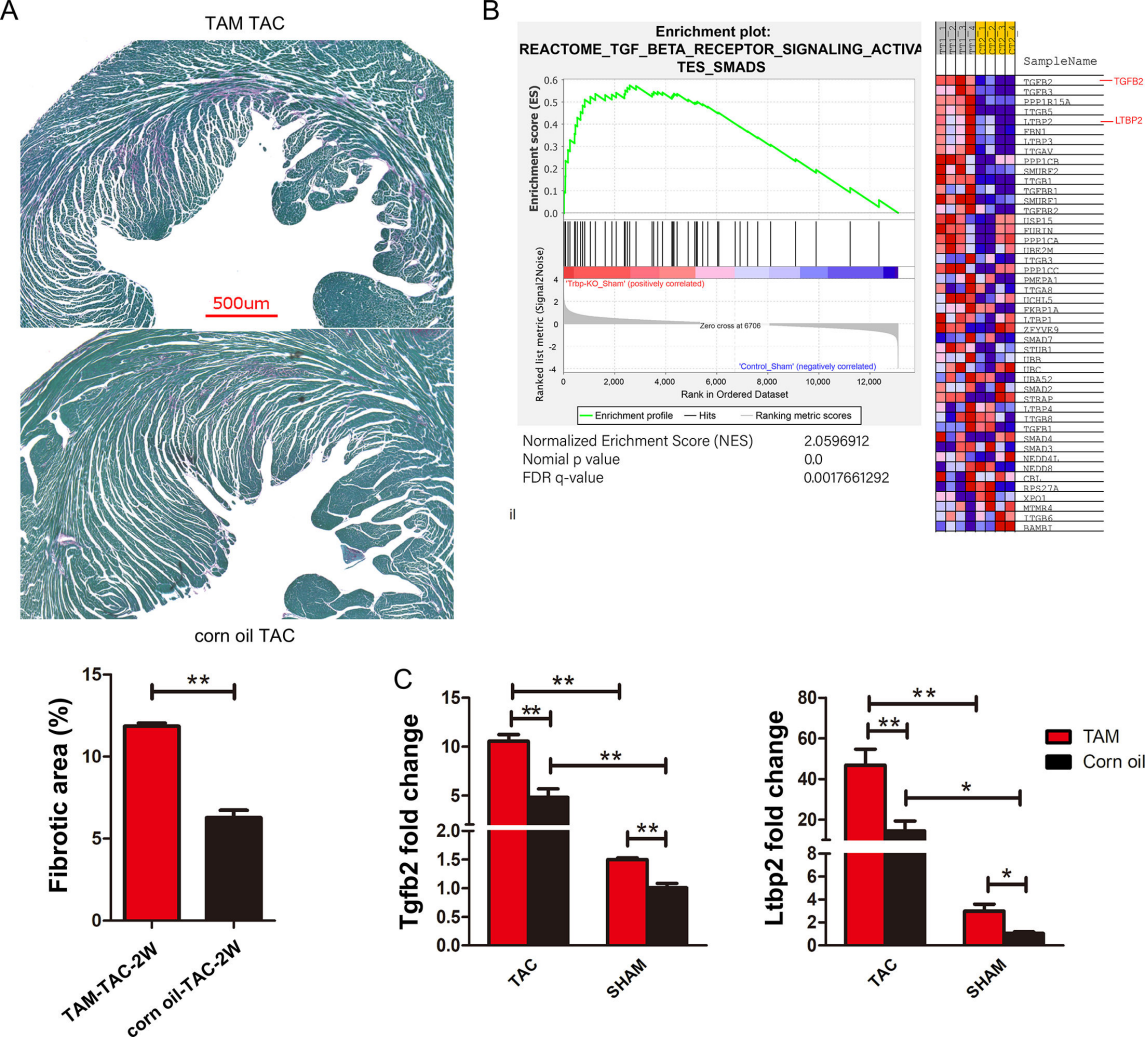
Activation of the TGF-β pathway is intensified in Trbp-cKO hearts prior to and after TAC-induced cardiac stress. **(A**) Representative images of cardiac sections from Trbp-cKO and Ctrl mouse hearts two weeks after TAC surgery stained with Fast Green to label fibrotic tissue. Scale bar = 500 µm. Quantification of fibrosis measured as percentage of total myocardial area in Trbp-cKO compared with control hearts following TAC (*n* = 3). (**B)** GSEA pathway analysis comparing Trbp-TAC and control-TAC groups. TC, Trbp-cKO TAC, CT, control (corn oil) TAC. (**C)** qRT-PCR analysis to determine *Tgfβ2* and *Ltbp2* expression levels in Trbp-cKO and control hearts eight weeks after either SHAM or TAC surgery (*n* = 4). *P < 0.05; **P < 0.01. Values are expressed as mean ± SD. Statistical differences between groups were examined by unpaired Student’s *t*-test. cKO, conditional knockout; TAC, transverse aortic constriction; TAM, tamoxifen.

While increased expression of genes associated with fibrosis is commonly observed in failing hearts [19], our data indicate changes in these genes prior to TAC stress, prior to the development of obvious cardiac fibrosis. We decided to refine our analysis and examine hearts for cardiac fibrosis two weeks after TAC. At this timepoint, Trbp-cKO mice display no significant difference in the cardiac function when compared with that of controls, and overt heart failure has not developed. In Trbp-cKO hearts, compared with hearts from control TAC mice, significant differences were seen in cardiac fibrosis two weeks after TAC (Figure 3A). These findings indicate that the amplified expression of fibrotic genes in the Trbp-cKO mouse hearts may accelerate cardiac fibrosis and contribute to the cardiac phenotype.

Our bioinformatic data indicate that biosynthesis and formation of collagen fibrils and other ECM-related enzymes are significantly activated (Supplementary Figure 3A-C). The TGF-β signaling pathway is a well-characterized regulator of fibrosis [20]. Our sequence data also indicate that after the induction of pressure overload in the heart, Trbp loss could significantly increase TGF-β pathway signaling (Figure 3B). We then verified that *Tgfβ2* and *Ltbp2*, two upstream genes of TGF-β pathway, were significantly increased in Trbp cKO-TAC hearts (compared with control TAC hearts; Figure 3C). In the absence of TAC stress, Trbp loss similarly induced both *Tgfβ2* and *Ltbp2* gene expression in mature cardiomyocytes (Figure 3C).

### Cardiomyocyte expressed Trbp regulates fibrosis gene expression in fibroblasts through cardiomyocyte–fibroblast cross-talk

**Figure 4:**
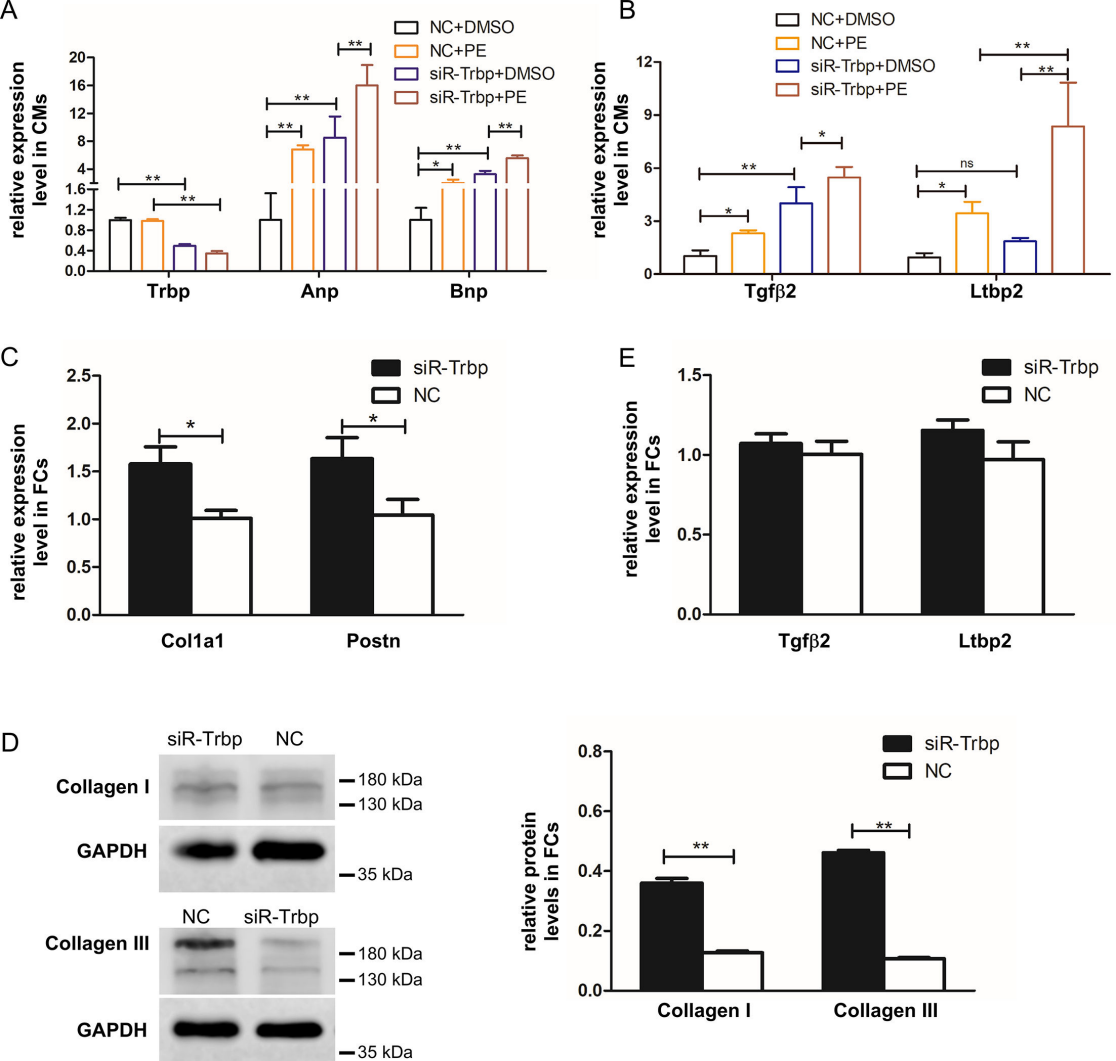
Knockdown of Trbp in NRVMs induces the expression of fibrotic markers in co-cultured NRCFs. **(A**) qRT-PCR analysis to determine levels of Trbp, Anp and Bnp transcripts in NRVMs with normal or decreased Trbp expression, with and without PE treatment. *n* = 3. (**B)** qRT-PCR analysis to determine levels of *Tgfβ2* and *Ltbp2* in NRVMs with normal or decreased Trbp expression, with and without PE treatment. *n* = 3. (**C)** qRT-PCR analysis to determine levels of *Col1a1* and *Postn* in NRFCs co-cultured with siR-Trbp or NC-treated NRVMs. *n* = 4. (**D)** Western blot analysis to determine levels of Collagen I and Collagen III in NRCFs after co-culture with NRVMs expressing normal or decreased levels of Trbp. *n* = 3. (**E)** qRT-PCR analysis to determine levels of *Tgfβ2* and *Ltbp2* in NRFCs co-cultured with siR-Trbp or NC-treated NRVMs. *n* = 4. *P < 0.05, **P < 0.01. Values are expressed as mean ± SD. Statistical differences between groups were examined by unpaired Student’s *t*-test. NC, negative control; NRCFs, neonatal rat cardiac fibroblasts; NRVMs, neonatal rat ventricle myocytes; PE, phenylephrine.

The finding that the loss of Trbp in cardiomyocytes resulted in a significant increase in fibrosis and the induction of the TGF-β pathway in adult hearts prompted us to hypothesize that Trbp-null cardiomyocytes communicate with neighboring fibroblasts to induce fibrosis. To test this hypothesis, we conducted co-culture experiments using isolated NRVMs and NRCFs [19]. We achieved consistent Trbp knockdown in NRVMs (Figure 4A) and found that *Tgfβ2* and *Ltpb2* expression increased in NRVMs with diminished Trbp under co-culture conditions (Figure 4B). We also examined the levels of hypertrophic markers and observed an increase in both Anp and Bnp transcript levels upon Trbp knockdown (Figure 4A). This change in hypertrophic marker levels was comparable to the increases observed when NRVMs were treated with phenylephrine (PE), an alpha-1 adrenergic receptor agonist which is known to induce cardiomyocyte hypertrophy [21] (Figure 4A). When PE treatment was combined with Trbp knockdown, the increases in hypertrophic marker gene expression, and in *Tgfβ2* and *Ltpb2* expression, were amplified (Figure 4A and B). We also observed an increase in the expression of the extracellular matrix gene *Col1a1*, as well as the proliferation marker *Postn*, in NRCFs that were co-cultured with NRVMs with reduced Trbp (i.e., untreated NRCFs with siTrbp-treated NRVMs; Figure 4C). The increase in collagen protein levels was confirmed by Western blot analysis of both Collagen I and Collagen III levels (Figure 4D). However, we did not observe significant changes in the transcript levels of *Tgfβ2* and *Ltpb2* in NRCFs upon co-culture with NRVMs with or without Trbp knockdown (Figure 4E); these data support a role for Trbp in the mediation of cross-talk between cardiomyocytes and fibroblasts by the TGF-β pathway.

**Figure 5:**
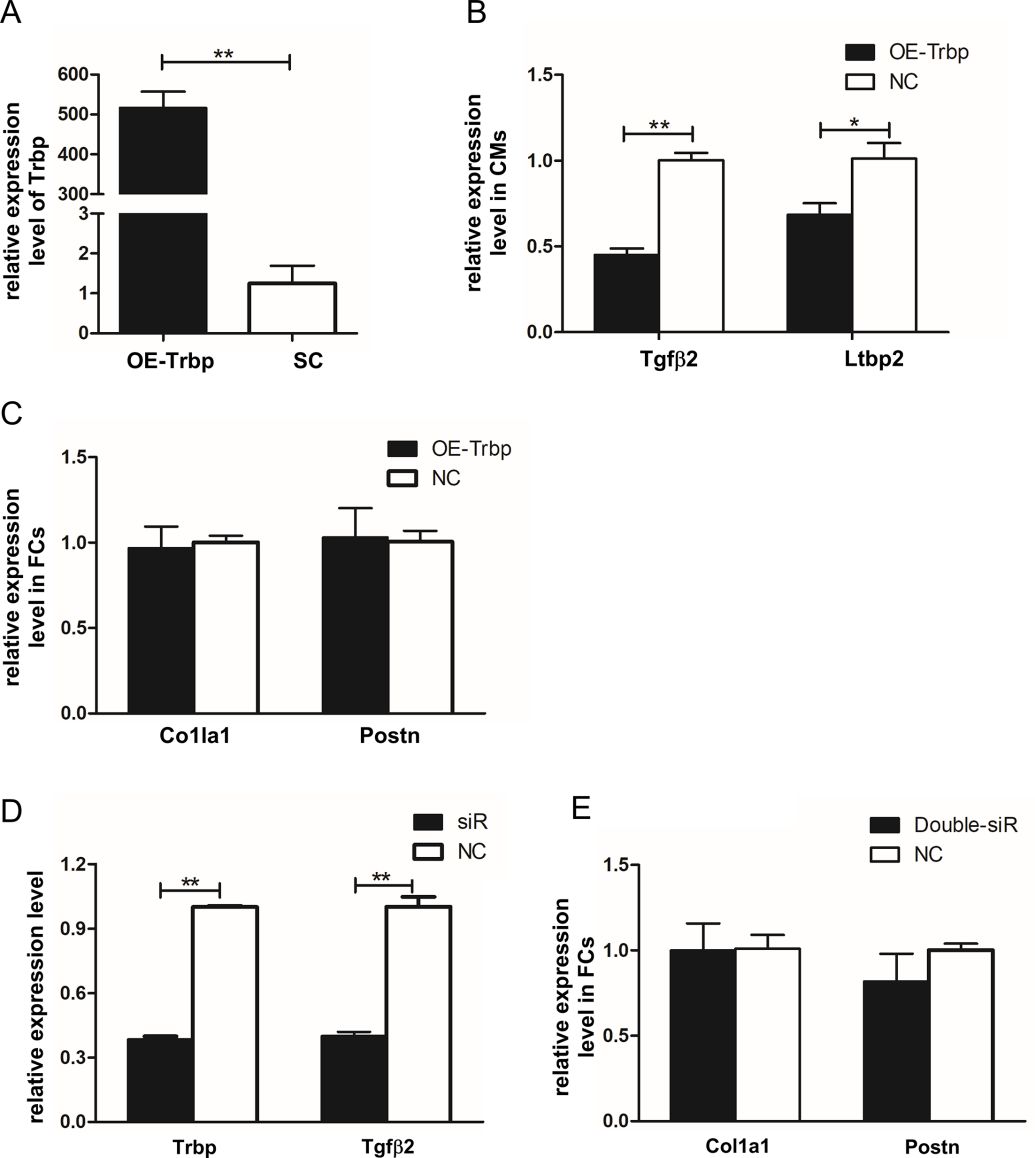
Analysis of the role of Trbp on the regulation of *Tgfβ2* and *Ltbp2* mRNA levels in NRVMs and the effect of altered Trbp and *Tgfβ2* levels on fibrotic gene expression in co-cultured NRCFs. **(A**) qRT-PCR analysis of Trbp expression in control and Trbp-OE NRVMs. (**B)** qRT-PCR analysis of *Tgfβ2* and *Ltbp2* expression in NRVMs with normal or elevated levels of Trbp. (**C)** qRT-PCR analysis of *Col1a1* and *Postn* expression in NRFCs co-cultured with NRVMs expressing normal or elevated levels of Trbp. (**D)** qRT-PCR analysis of Trbp and *Tgfβ2* levels in control, siR-Trbp and siR-Tgfβ2-treated NRVMs. (**E)**qRT-PCR analysis of *Col1a1* and *Postn* levels in NRFCs co-cultured with NRVMs having normal or reduced expression of both Trbp and *Tgfβ2*. Panels A, B, C, and E, *n* = 4. Panel D, NC, *n* = 3; siR, *n* = 4. *P < 0.05, **P < 0.01.Values are expressed as mean ± SD. Statistical differences between groups were examined by unpaired Student’s *t*-test. OE, overexpression; NC, negative control; NRCFs, neonatal rat cardiac fibroblasts; NRVMs, neonatal rat ventricle myocytes.

Next, we asked whether Trbp overexpression (OE) in cardiomyocytes could affect the TGF-β pathway and the fibrotic gene expression in fibroblasts. We employed adenovirus to overexpress Trbp as previously described [22] and confirmed the increased expression of Trbp after the infection of NCVMs (Figure 5A). We found that Trbp-OE down-regulated both *Tgfβ2* and *Ltbp2* in NRVMs (Figure 5B). However, there is no significant change of fibrotic markers in NRCFs, with or without co-culture with Trbp-OE NRVMs (Figure 5C).

### Inhibiting TGF-β pathway suppresses fibrosis resulted from Trbp knockdown

The data presented above demonstrate that the inhibition of Trbp in cardiomyocytes induces the expression of a fibrotic gene profile in fibroblasts, at least in part due to the induction of the TGF-β pathway. We next examined whether the inhibition of the TGF-β pathway could circumvent this cardiomyocyte-based Trbp-mediated regulation of fibrotic gene expression. *Tgfβ2* is substantially induced in Trbp-inhibited cardiomyocytes (Figure 4B), as well as that of the hearts of Trbp-cKO mice, with or without TAC procedure (Figure 3C). We successfully knocked down *Tgfβ2* in Trbp-siR NRVMs (Figure 5D); we then co-cultured these NRVMs having diminished Trbp and *Tgfβ2* expression with NRCFs. As expected, the expression of the fibrotic markers *Col1a1* and *Postn* was not inducted in the NRCFs (Figure 5E). This result further supports the role of Trbp inhibition in cardiomyocytes in the TGF-β pathway-dependent induction of fibrosis.

## Discussion

In this study, we provide evidence that Trbp is an important regulator of the cross-talk between cardiomyocytes and fibroblasts. This function is more pronounced in response to pressure overload-induced remodeling of the heart. This new function for Trbp expands upon its previously characterized role in the regulation of fast- and slow-twitch myofiber gene expression during heart development and postnatal growth [8] and establishes another important role for this protein in the mature heart. Cardiomyocyte-specific Trbp deletion slightly affected baseline function of the heart in young adult mice but did not promote cardiac remodeling, assessed three months after Trbp cKO. However, even in these animals, we observed an increase in the expression of fibrotic genes (e.g., *Col1a1*, *Col3a1,* and *Postn*); we provide evidence that this induction in a fibrotic gene expression profile of Trbp-cKO hearts is mediated through cross-talk between cardiomyocytes and fibroblasts. Further stressing the heart by surgical intervention (TAC) augmented the level of fibrotic gene expression and caused more severe cardiac fibrosis and worsened heart function. These data indicate a potential protective role for Trbp in the mature heart exposed to pressure overload conditions.

Trbp has two very well-characterized functions in cellular regulation [5,23,24]: (1) regulation of microRNA (miRNA) biogenesis through interaction with Dicer and (2) inhibition of protein kinase R function by dsRNA sequestration and by direct protein–protein interaction during virus pathogenesis. Our previous study demonstrated that Trbp regulates fast- and slow-twitch myofiber gene expression through miRNA-mediated Sox6 repression [8]. Therefore, when we conducted this study to explore the role of Trbp in the mature heart, we expected to find the roles for miRNAs in linking Trbp function to fibrotic gene expression. We did observe a significant decrease in miR208a expression after conditional deletion of Trbp; following TAC, its expression further decreased in Trbp-CKO hearts (Supplementary Figure 4A). By using miR target scan databases (https://mirdb.org and https://mirbase.org), we filtered the list of differentially expressed genes generated by our RNA-seq analysis for potential miR208a target genes. Sox6 was increased in Trbp-cKO hearts but was not further induced following TAC, suggesting that Sox6 is not responsive to pressure overload (Supplementary Figure 4B).

We also found that the expression of genes encoding secreted proteins with previously reported roles in ECM formation and remodeling was significantly increased in Trbp cKO hearts (i.e., *Tgfβ2* and *Ltbp2*). Also, fibrotic markers gene expression was up-regulated (e.g., *Col1a1*, *Col3a1*, and *Postn*). This increase in expression of fibrosis-related genes was amplified following TAC in Trbp-cKO animals. Although the expression of these genes was also up-regulated in the sham Trpb cKO heart as noted, only in the presence of pressure overload does the crucial role of Trpb in regulating fibrosis become apparent. This led us to examine the TGF-β pathway as a possible mediator of the elevated fibrotic gene expression and subsequent accelerated cardiac remodeling and worsened the heart function in Trbp cKO mice following TAC. We compared our RNA-seq and small RNA-seq (derived from our previous analysis of cardiac tissue from hearts of embryos with early cardiac deletion of Trbp; GSE67658) datasets; the small RNA-seq data from our previous study demonstrated that miRNA expression in one-month-old Trbp-cKO hearts was distinct from the expression patterns observed in adult hearts [25]. We initially identified several miRNAs as potential links to the TGF-β pathway; unfortunately, we failed to identify any miRNA that demonstrated a change in its ability to regulate fibrotic gene expression in response to Trbp deletion (data not shown).

Our *in vitro* studies demonstrated a role for Trbp in the regulation of TGF-β pathway gene expression. TGF-β is secreted from cells as a multiprotein complex that is covalently bound to LTBP1, LTBP3, and LTBP4. These proteins target the latent complex to sites within the ECM for storage where it awaits activation [26–28]. A recent study demonstrated that increased expression of LTBP2 was associated with a more pronounced localization of activated fibroblasts to regions of fibrosis within myocardial tissue [29]. The importance of cross-talk between cardiomyocytes and fibroblasts has been reported previously [19,30,31]. Some of these studies have described important functions for small RNAs and secreted proteins; these include the role of fibroblast-secreted miR21 in the induction of cardiomyocyte hypertrophy [31] and the ability of miR30d, secreted by cardiomyocytes, to promote fibroblast activation [19]. Our data expands on these studies to demonstrate how cardiomyocyte derived Trbp can regulate fibroblasts through the inhibition of *Tgfβ2* and *Ltbp2* expression; these data suggest an important new avenue for the therapeutic inhibition of cardiac fibrosis.

**Clinical Perspectives**

- The mechanisms underlying the response of the heart to stress remain poorly understood. Pathophysiological factors, often with a genetic basis, can induce cardiac remodeling that results from changes in cellular signaling pathways and intercellular communication.
- In this report, we demonstrate that Trbp plays a critical role in regulating cardiac fibrosis during cardiac remodeling by mediating the cross-talk between cardiomyocytes and fibroblasts through the TGF-β signaling pathway.
- Our findings highlight the critical role of Trbp in adult hearts, suggesting that it may serve as a novel therapeutic target to prevent the fibrosis associated with heart disease and help improve cardiac function in failing hearts.

## Supplementary material

Online Supplementary Figures 1 to 4

Online Supplementary Tables 1 to 3

Online Supplemental Table 4

## Data Availability

All data and methods used in this analysis will be made available to researchers upon request. Next generation sequencing data have been deposited in the Gene Expression Omnibus (GEO) database under the accession code GSE287292.

## Abbreviations

Col1a1: collagen type I alpha 1 chain
Col3a1: collagen type III alpha 1 chain
DMEM: Dulbecco's Modified Eagle Medium
IFN: Interferon
LAP: latency-associated peptide
LTBP: latent transforming growth factor beta binding protein
LVEF: left ventricular ejection fraction
LVFS: left ventricular fractional shortening
MMLV: Moloney Murine Leukemia Virus
MerCreMer: tamoxifen inducible Cre recombinase
NRCFs: neonatal rat cardiac fibroblasts
NRCMs: cultured neonatal rat ventricle cardiomyocytes
OE: overexpressing
Postn: periostin
RBP: RNA-binding protein
Sox6: SRY-box transcription factor 6
TAC: transverse aortic constriction
TGF-β: transforming growth factor beta
Trbp: TARBP2 subunit of RISC loading complex
cKO: conditional knockout
cTNT: troponin T2, cardiac type
αMHC: alpha myosin heavy chain
