# Supplementary material for: Trbp inhibits cardiac fibrosis through TGF-β pathway-mediated cross-talk between cardiomyocytes and fibroblasts
Source: Clin Sci (Lond). 2025 Mar 11;139(5):325–38. doi: 10.1042/CS20242397 (PMC12203991; doi:10.1042/CS20242397)
Supplement: Online Supplementary Figures 1 to 4 [file CS-139-05-CS20242397-s001.pdf]

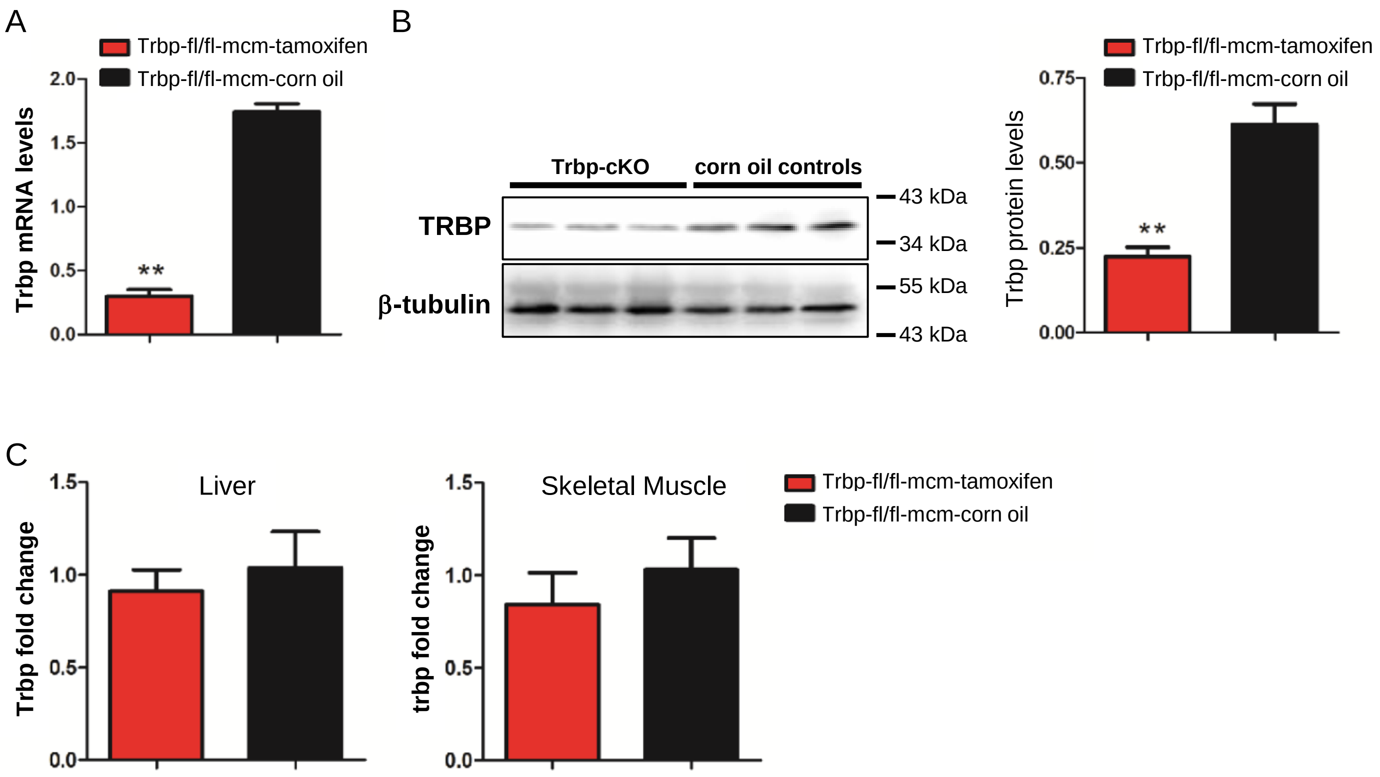

Supplementary Figure 1: Confirmation of cardiac specific reduction of Trbp in Trbp-cKO mice. A, qRT-PCR analysis of Trbp levels in Trbp-cKO hearts 1 week after either corn oil or TAM injection. **B,** Western blot analysis to determine levels of Trbp protein in Trbp-cKO hearts 1 week after either corn oil or TAM injection. β-tubulin is included as a loading control. **C,** qRT-PCR analysis of Trbp levels in liver and skeletal muscle tissue from Trbp-cKO mice 1 week after either corn oil or TAM injection. The generation of the Trbp-cKO line is described in the Materials and Methods section. Panel A, B and C, n= 3, **P<0.01 compared with controls. Values are expressed as mean ± SD. Statistical differences between groups were examined by unpaired Student's t-test.

A

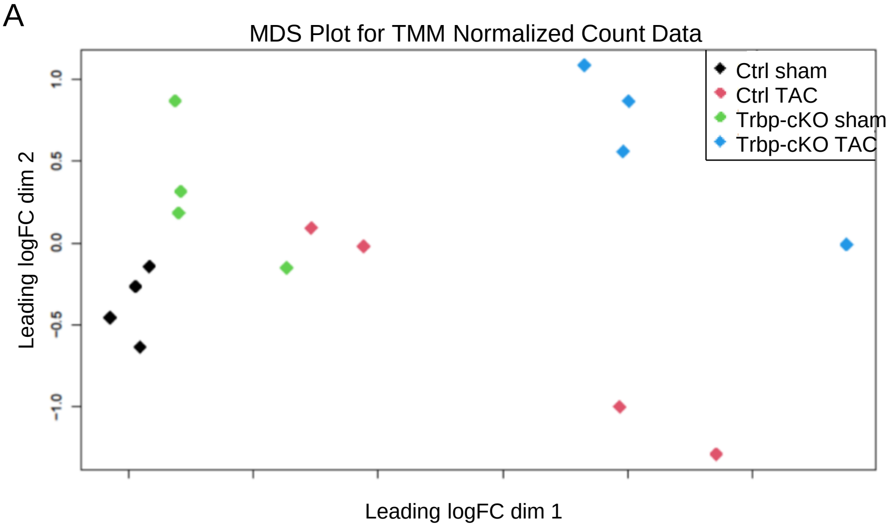

B

Name	Total	Unique	_Color_
CT_v_CS_DN	348	348	#E41A1C
CT_v_CS_UP	585	585	#377EB8
TS_v_CS_DN	106	106	#4DAF4A
TS_v_CS_UP	152	152	#984EA3
TT_v_CT_DN	202	202	#FF7F00
TT_v_CT_UP	320	320	#FFFF33
TT_v_TS_DN	436	436	#A65628
TT_v_TS_UP	595	595	#F781BF

C

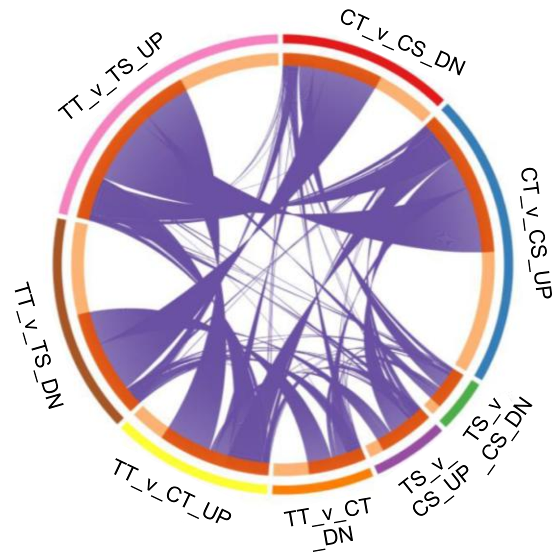

Supplementary Figure 2: RNA-seq analysis reveals expression changes in fibrotic and TGF-β signaling pathways in Trbp-cKO mouse hearts. A, RNA-seq analysis was performed on tissue collected from four groups: control and Trbp-cKO mice subjected to either sham or TAC surgery. The MDS plot of the four groups demonstrate clear segregation between them. **B,** Differentially expressed genes (DEG) between the four experimental groups were compared. The DEG comparisons are illustrated after filtering as described in the Materials and Methods section. **C,** Chord diagram illustrating significant linkage between genes in different groups. Concordance between genes regulated in response to Trbp expression change or TAC intervention are indicated.

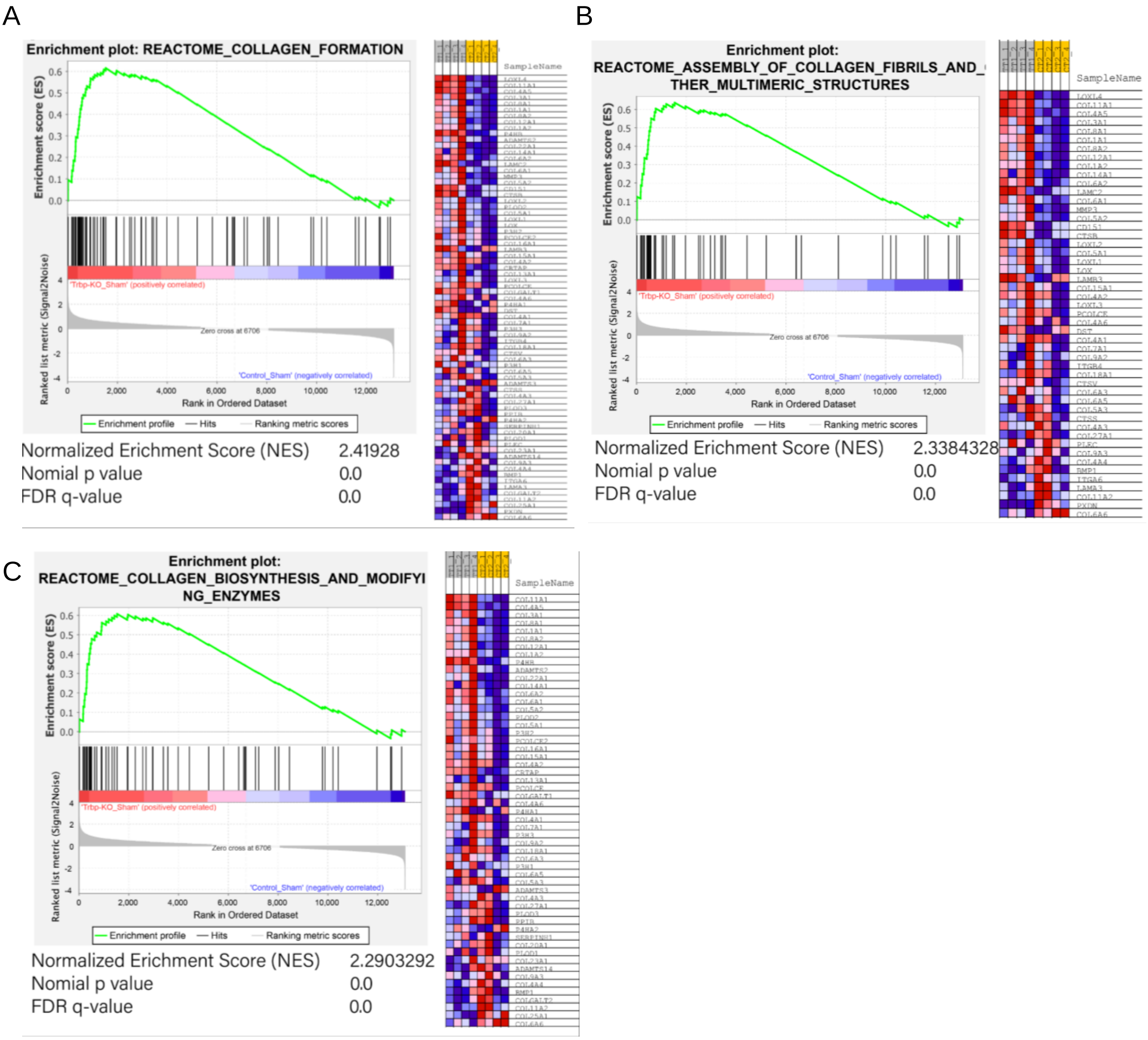

Supplementary Figure 3: Comparison of gene expression for pathways regulating collagen biosynthesis and formation, and ECM related enzyme function. A, Enrichment plot demonstrating increased expression of genes associated with collagen formation. **B,** Enrichment plot demonstrating increased expression of genes associated with assembly of collagen fibrils and other multimeric structures. **C,** Enrichment plot demonstrating increased expression of genes associated with collagen biosynthesis and associated modifying enzymes. All analyses were performed by comparing Trbp-cKO-TAC hearts with control TAC hearts.

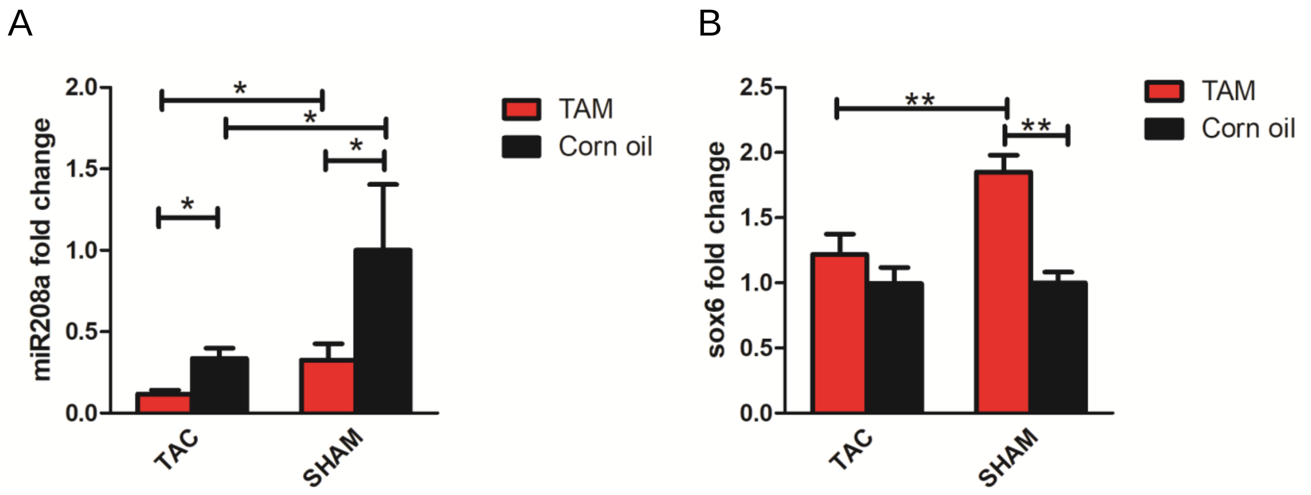

Supplementary Figure 4: Analysis of miR208a and Sox6 expression levels in Trbp-cKO mouse hearts in response to pressure overload. A, qRT-PCR analysis of miR208a expression in Trbp-cKO hearts after sham or TAC surgery. TAM TAC, n=4; corn-oil TAC, n=4; TAM SHAM, n=3; corn-oil SHAM, n=3. **B,** qRT-PCR analysis of Sox6 expression in Trbp-cKO hearts after sham or TAC surgery (TAM TAC, n=7; corn-oil TAC, n=6; TAM SHAM, n=5; corn-oil SHAM, n=5. *P<0.05 **P<0.01. The values are expressed as the mean fold change ± SD (relative to the corn oil sham control). Statistical differences between groups were examined by unpaired Student's t-test.
