# Supplementary material for: Trbp inhibits cardiac fibrosis through TGF-β pathway-mediated cross-talk between cardiomyocytes and fibroblasts
Source: Clin Sci (Lond). 2025 Mar 11;139(5):325–38. doi: 10.1042/CS20242397 (PMC12203991; doi:10.1042/CS20242397)
Supplement: Online Supplementary Tables 1 to 3 [file CS-139-05-CS20242397-s002.pdf]

	Trbp-fl/fl-mcm-Tamoxifen n=14	Trbp-fl-fl-mcm-corn oil n=10	Trbp-fl/fl-Tamoxifen n=5	Trbp-fl/fl-corn oil n=5
HR (bpm)	569 ± 17	527 ± 21	517 ± 22	531 ± 22
BW (g)	26.08 ± 0.63	28.83 ± 0.87	26.66 ± 1.03	26.80 ± 1.46
**LV end diastole**				
IVS (mm)	0.71 ± 0.03	0.78 ± 0.02	0.80 ± 0.03	0.73 ± 0.01
LVID (mm)	3.43 ± 0.06	3.31 ± 0.08	3.65 ± 0.14	3.41 ± 0.12
LVPW (mm)	0.78 ± 0.02	0.80 ± 0.03	0.76 ± 0.02	0.80 ± 0.02
LV volume (µl)	48.99 ± 1.95	44.95 ± 2.66	50.66 ± 3.77	48.16 ± 3.85
**LV end systole**				
IVS (mm)	1.21 ± 0.04	1.27 ± 0.04	1.28 ± 0.04	1.22 ± 0.0.03
LVID (mm)	2.00 ± 0.05	1.87 ± 0.06	2.10 ± 0.13	1.81 ± 0.09
LVPW (mm)	1.20 ± 0.02	1.17 ± 0.01	1.16 ± 0.02	1.24 ± 0.02
LV volume (µl)	12.90 ± 0.76	10.90 ± 2.91	13.10 ± 1.97	10.05 ± 1.28
LVEF %	73.83 ± 1.38	75.66 ± 1.37	74.08 ± 2.98	79.17 ± 1.71
LVFS %	42.02 ± 1.15	43.49 ± 1.32	42.51 ± 2.54	46.90 ± 1.76
LV MASS (mg)	65.36 ± 2.67	67.73 ± 3.83	69.74 ± 3.73	68.68 ± 5.23

Supplementary Table 1: Evaluation of cardiac function in mice 3 weeks after TAM injection. Parameters for cardiac function were determined by echocardiography at 3 weeks post tamoxifen or corn oil injection into Trbp-fl/fl-mcm (i.e., Trbp-cKO) or Trbp-fl/fl mice. N values are indicated for each group on the table. HR, heart rate; BW, body weight; IVS, interventricular septal; LVID, left ventricle internal dimension; LVPW, left ventricle posterior wall thickness. Values are expressed as mean ± SD.

	Trbp-fl/fl-mcm-Tamoxifen<br>n=6	Trbp-fl-fl-mcm-corn oil<br>n=4
HR (bpm)	579 ± 17	536 ± 11
BW (g)	32.30 ± 1.46	31.85 ± 0.94
**LV end diastole**		
IVS (mm)	0.79 ± 0.01	0.78 ± 0.01
LVID (mm)	3.67 ± 0.03*	3.49 ± 0.05
LVPW (mm)	0.78 ± 0.01	0.80 ± 0.01
LV volume (µl)	56.81 ± 1.44	50.67 ± 1.67
**LV end systole**		
IVS (mm)	1.24 ± 0.01	1.23 ± 0.03
LVID (mm)	2.23 ± 0.05*	2.01 ± 0.06
LVPW (mm)	1.23 ± 0.01	1.20 ± 0.01
LV volume (µl)	17.84 ± 1.03	12.84 ± 0.89
LVEF %	68.68 ± 1.25*	74.49 ± 2.02
LVFS %	37.75 ± 0.98*	42.55 ± 1.86
LV MASS (mg)	79.70 ± 1.96	74.10 ± 1.56

Supplementary Table 2: Evaluation of cardiac function in mice 6 months after TAM injection. Parameters for cardiac function were determined by echocardiography at 6 months post tamoxifen or corn oil injection into Trbp-fl/fl-mcm (i.e., Trbp-cKO) mice. N values are indicated for each group on the table. HR, heart rate; BW, body weight; IVS, interventricular septal; LVID, left ventricle internal dimension; LVPW, left ventricle posterior wall thickness. *compared with controls, $P<0.05$. Values are expressed as mean ± SD. Statistical differences between groups were examined by unpaired Student's t-test.

Mouse qRT-PCR Primers

Trbp-qF	AGGAGGGAATGAGTGAAGAGG
Trbp-qR	GAAGGCTGATCGGGGTCT
Sox6-qF	TTACAACCACAGACAGATTGAGC
Sox6-qR	TGGCTGTGGAGTTGATGG
Myh7b-qF	GCACGGAGCTCAAGAAAGAC
Myh7b-qR	GCCATCTCTTCTGTCAGGTTCT
Myl3-qF	AAGAAGGATGATGCCAAAGC
Myl3-qR	GCTCAGGCTCTGGTGCAG
Myl9-qF	GATAAGGAGGACCTGCACGA
Myl9-qR	GCCCTCCAGATACTCGTCTG
Tpm2-qF	CGAGGTGGCTGAGAGTAAATG
Tpm2-qR	TCCAGGGATTTCAAGTTGTTG
Tnnc1-qF	CGACAGCAAAGGGAAGTCTG
Tnnc1-qR	TGTAGCCATCAGCGTTTTTG
ANP-qF	TACAGTGCGGTGTCCAACACAG
ANP-qR	TGCTTCCTCAGTCTGCTCACTC
BNP-qF	TCCTAGCCAGTCTCCAGAGCAA
BNP-qR	GGTCCTTCAAGAGCTGTCTCTG
Tgfb2-qF	CCCCACATCTCCTGCTAATG
Tgfb2-qR	TCAATGTAAAGAGGGCGAAGG
Ltbp2-qF	AACAGCACCAACCACTGTATC
Ltbp2-qR	CCTGGCATTCTGAGGGTCAAA
GAPDH-qF	GGCAAGTTCAATGGCACAGT
GAPDH-qR	TGGTGAAGACGCCAGTAGACTC

Rat qRT-PCR Primers

Trbp-qF	GAGCGTTTCATTGAGATTGG
Trbp-qR	CCCACGGTTCCTTAGTCC
ANP-qF	GCTCGAGCAGATCGCAAAAG
ANP-qR	GAGTGGGAGAGGTAAGGCCT
BNP-qF	GACGGGCTGAGGTTGTTTTA
BNP-qR	ACTGTGGCAAGTTTGTGCTG
Tgfb2-qF	CGGAGGTGATTTCCATCTACAACAG
Tgfb2-qR	TGGGCAGACAGTTTCGGAGG
Ltbp2-qF	CGGGGTCCTTCTTCTGTCTC
Ltbp2-qR	CACACTCTCCACTGTCTGGG
Col1a1-qF	AAGGTGACAGAGGCATAAAGGG
Col1a1-qR	AGGGAGACCGTTGAGTCCAT
Postn-qF	TATCACAGGAGGAGCGGTGT
Postn-qR	AAGACGCCAACCCTAACTGG
GAPDH-qF	GACATGCCGCCTGGAGAAAC
GAPDH-qR	CTGTGGTGCTTTGAGGTAGGTC

Supplementary Table 3: PCR primers used for quantitative PCR (qPCR) analyses. The mouse and rat primer sets used for the qPCR assays are provided. The primer name is prefaced by the gene name. qF, qPCR forward primer; qR, qPCR reverse primer.
